# Epigenetic age deacceleration in youth at familial risk for schizophrenia and bipolar disorder

**DOI:** 10.1038/s41398-023-02463-w

**Published:** 2023-05-08

**Authors:** Alex G. Segura, Elena de la Serna, Gisela Sugranyes, Inmaculada Baeza, Isabel Valli, Covadonga Díaz-Caneja, Nuria Martín, Dolores M. Moreno, Patricia Gassó, Natalia Rodriguez, Sergi Mas, Josefina Castro-Fornieles

**Affiliations:** 1 Department of Clinical Foundations, Pharmacology Unit, University of Barcelona, Barcelona, Spain; 2 Child and Adolescent Psychiatry and Psychology Department, 2021SGR01319, Institute of Neuroscience, Hospital Clínic de Barcelona, Barcelona, Spain; 3 Department of Medicine, Institute of Neuroscience, University of Barcelona, Barcelona, Spain; 4 Centro de Investigación Biomédica en Red de Salud Mental (CIBERSAM), Madrid, Spain; 5 Institut d’Investigacions Biomèdiques August Pi i Sunyer (IDIBAPS), Barcelona, Spain; 6 Department of Child and Adolescent Psychiatry, Hospital General Universitario Gregorio Marañón, Madrid, Spain; 7 Adolescent Inpatient Unit, Department of Psychiatry, Hospital General Universitario Gregorio Marañón, Madrid, Spain; 8 Psychiatry Department, Universidad Complutense de Madrid, Madrid, Spain

**Keywords:** Personalized medicine, Clinical genetics

## Abstract

Epigenetic modifications occur sequentially during the lifespan, but their pace can be altered by external stimuli. The onset of schizophrenia and bipolar disorder is critically modulated by stressors that may alter the epigenetic pattern, a putative signature marker of exposure to environmental risk factors. In this study, we estimated the age-related epigenetic modifications to assess the differences between young individuals at familial high risk (FHR) and controls and their association with environmental stressors. The sample included 117 individuals (6–17 years) at FHR (45%) and a control group (55%). Blood and saliva samples were used estimate the epigenetic age with six epigenetic clocks through methylation data. Environmental risk was measured with obstetric complications, socioeconomic statuses and recent stressful life events data. Epigenetic age was correlated with chronological age. FHR individuals showed epigenetic age deacceleration of Horvath and Hannum epigenetic clocks compared to controls. No effect of the environmental risk factors on the epigenetic age acceleration could be detected. Epigenetic age acceleration adjusted by cell counts showed that the FHR group was deaccelerated also with the PedBE epigenetic clock. Epigenetic age asynchronicities were found in the young at high risk, suggesting that offspring of affected parents follow a slower pace of biological aging than the control group. It still remains unclear which environmental stressors orchestrate the changes in the methylation pattern. Further studies are needed to better characterize the molecular impact of environmental stressors before illness onset, which could be critical in the development of tools for personalized psychiatry.

## Introduction

Schizophrenia and bipolar disorder are impairing conditions that have differential diagnostic criteria. However, their familial aggregation and overlapping clinical and genetic features do not fully correlate with their nosological boundaries, pointing towards a partially shared etiology [1–4]. The individuals at familial high risk (FHR) have a two- to fourfold increase in the risk of developing a psychiatric disorder, for which the exposure to environmental stressors have a critical role [5].

Schizophrenia and bipolar disorder are associated with a shorter lifespan, which has been linked to age-related biomarkers and physiological conditions such as increased inflammation and oxidative stress, a shorter telomere length and metabolic disruption [6–13], suggesting that patients suffer from the effects of accelerated aging. Epigenetic modifications (changes in chromatin structure, primarily measured by assessing the methylation of CpG dinucleotides) have been closely related to gene expression, driving cell senescence and affecting their function [14]. Methylation patterns change throughout the lifespan, following a specific timing. Epigenetic clocks measure the methylation of specific sets of CpGs for the estimation the epigenetic age in years, a proxy of the biological age of the individual. Epigenetic age correlations with chronological ages of schizophrenia patients and the direction of these are inconsistent and vary across epigenetic clocks [15, 16].

Schizophrenia and bipolar disorder prediction models perform best when including polygenic constructs, multiple environmental factors and their interaction [17]. The characterization of risk factors encompasses multiple sorts of environmental impacts occurring throughout all stages of life [18, 19], which can lead to an acceleration the epigenetic age [16]. Obstetric complications, including maternal and perinatal infections that drive immune responses in the offspring, are thought to cause a neurodevelopmental disruption [20, 21]. Early life adversity (ranging from explicit violence to subtle forms of emotional negligence) has been associated with more severe manifestations of the disorders and suicidal behaviors [22–24]. Moreover, recent traumatic events may also be a substantial risk factor for disorder onset [25, 26]. Young individuals experiencing migration processes, lower socioeconomic statuses and urbanicity—linked to social exclusion and isolation—are also at a higher risk of developing schizophrenia [27–29].

In this study, we examined the epigenetic age of a sample consisting of FHR individuals and a control group. Blood and saliva samples were used to estimate their epigenetic age using six epigenetic clocks. We expected that the FHR group would report greater asynchronicities between their epigenetic and chronological age than the control group. Furthermore, we believed that these differences would be associated with the exposure to environmental stressors.

## Methods

The present study is part of the Bipolar and Schizophrenia Young Offspring Study (BASYS), which is a multicenter, longitudinal, naturalistic study that aims to compare the clinical, neuropsychological, neuroimaging, genetic and epigenetic characteristics of the child and adolescent offspring of patients diagnosed with SZ or BD and of a community control group. This study was conducted in the child and adolescent psychiatry units of two hospitals in Spain: the Hospital Clinic in Barcelona and Hospital Gregorio Marañón in Madrid. The methodology as well as the clinical and cognitive characteristics of the sample have been described previously in detail [30].

### Sample characteristics

The individuals at FHR were offspring of patients with schizophrenia or bipolar disorder, recruited by psychiatrists from the adult psychiatry units of both hospitals. The inclusion criteria were: (a) age between 6 and 17 years, and (b) a parent diagnosed with schizophrenia or bipolar disorder. The exclusion criteria were: (a) intellectual disability with an impact on functioning, and (b) significant head injury or a current medical or neurological condition. The only inclusion criterion for the offspring of the community controls was an age between 6 and 17 years, while the exclusion criteria were exactly the same as those for the FHR group plus a family history of psychotic disorders in first- or second-degree relatives. As this study focused on epigenetic data, only the individuals who had provided biological samples for DNA methylation analysis (53 FHR and 64 controls) were assessed.

### Ethical considerations

All procedures contributing to this work comply with the ethical standards of the relevant national and institutional committees on human experimentation and with the Helsinki Declaration of 1975, as revised in 2008. Written informed consent was obtained from one of the parents, having the other parent been informed, together with written assent from the participant if aged 12 and above.

### Clinical and environmental assessment

A trained psychiatrist or psychologist performed a mental health assessment of all the parents using the Spanish version of the Structured Clinical Interview for DSM-IV Disorders (SCID-I) [31, 32]. Parents or primary caregivers were also interviewed about their children. The study participants were assessed directly by trained child psychiatrists or psychologists who were blind to their parental diagnoses, using the Spanish version of the Schedule for Affective Disorders and Schizophrenia for School-Age Children - Present and Lifetime Version (K-SADS-PL) [33, 34].

Information about obstetric complications was collected using the Lewis-Murray scale [35]. This scale rates 15 obstetric complications as absent or definitely present, while 9 of the exposures can also be rated as equivocally present. For this study, history of obstetric complications was considered positive if at least one complication was definitely present.

The socioeconomic status was calculated according to the Hollingshead and Redlich scale [36]. The higher socioeconomic level between each set of parents was considered. The higher socioeconomic level between each set of parents was considered. Lower scores indicate a low socioeconomic status.

The occurrence of recent stressful events was determined using the Stressful Life Events Schedule (SLES), child-reported version [37, 38]. The SLES evaluates the presence/absence of a list of potentially stressful, age-adapted events in the last 12 months and rates their potential impact on a scale of 1 (not at all) to 4 (a lot). The SLES provides two scores: the number of stressful life events (SLEs) in the previous year and the score for the total cumulative impact of the SLEs.

### Biological samples

Blood samples were collected in EDTA tubes (K2EDTA BD Vacutainer EDTA tubes; Becton Dickinson, Franklin Lakes, NJ, USA) and genomic DNA was extracted with the MagNA Pure LC DNA Isolation Kit III and a MagNA Pure LC system (Roche Diagnostics GmbH, Mannheim, Germany). Saliva samples were collected using the Oragene DNA Saliva Collection Kit (OG-500, DNA Self-Collection Kit, Genotek, Ottawa, ON, Canada) and DNA was extracted according to the manufacturer’s instructions. DNA concentration and quality were measured spectrophotometrically using a NanoDrop 2000 spectrophotometer (Thermo Fisher Scientific, Epsom, Surrey, UK). DNA methylation *β*-values were obtained at GenomeScan using the Illumina Infinium MethylationEPIC BeadChip Kit.

### Methylation data collection

Raw intensity data (.IDAT) files were received and parallel bioinformatics processes were conducted in-house using the Chip Analysis Methylation Pipeline (ChAMP) Bioconductor package [39], which were performed separately for the methylation data obtained from blood samples (*n* = 79) and saliva samples (*n* = 38). Raw .IDAT files were used to load the data into the R environment with the *champ.load* function, which also enabled the simultaneous undertaking of the probe QC and removal steps. Probes with weak signals (*p* < 0.01), cross-reactive probes, non-CpG probes, probes with < 3 beads in at least 5% of the samples per probe, probes that bound to SNP sites, and sex chromosomes were all considered problematic for the accurate detection of downstream methylation and were therefore removed. *β*-values were then normalized using the *champ.norm* function, specifically with the beta-mixture quantile method (BMIQ function). Next, the singular value decomposition (SVD) method was performed with *champ.SVD* to assess the amount and significance of the technical batch components in our dataset. Using the *champ.runCombat* function, combat algorithms were applied to correct for slide and array (significant components detected by the SVD method).

### Epigenetic clock construction

The *methylclock* R package [40] was used to construct six epigenetic clocks. Horvath is a multi-tissue-based epigenetic clock designed to predict chronological age in individuals along the whole lifespan [41]. Similarly, Hannum and Wu epigenetic clocks estimate the epigenetic patterns linked to chronological age in blood tissues in adults and children, respectively [42, 43]. PedBE epigenetic clock was constructed for saliva samples in children [44]. Levine epigenetic clock captures the methylation patterns of “phenotypic aging”, mortality and morbidity epigenetic patterns rather than with chronological age [45]. CpGs located in telomeric regions can also be measured to estimate telomere length (TL), a well-established biomarker of health conditions associated with aging. The TL estimation by means of epigenetic markers used in this study was constructed with blood samples of adults [46].

Briefly, from normalized and batch-corrected methylation data, the package extracts the methylation levels of the available CpGs included in each clock. Subsequently, the coefficients obtained through an elastic net in the prediction models of each of the clocks in the original studies are used to predict the epigenetic age. Several studies have demonstrated that the epigenetic clocks are resistant to the CpG site missingness from the MethylationEPIC BeadChip Kit [47]. For each clock, we obtained the epigenetic age in years. Epigenetic age acceleration for every epigenetic clock was obtained after regressing chronological age on the epigenetic age. Cell-adjusted epigenetic age acceleration was obtained after regressing epigenetic age acceleration by seven cell-type proportions known to change throughout the lifespan, estimated differently for the blood [48] and saliva [49] samples.

### Statistical analysis

All the analyses were performed with R v4.1.2 [50]. Multiple testing correction was applied in all the analyses by means of the FDR method, and the threshold of significance of the two-sided adjusted *p* value (p.adj) was set at α < 0.05.

Group differences in sociodemographic features and environmental risk factors were calculated by linear mixed-effects models for continuous variables, using family relatedness as a random effect, and by chi square tests for categorical variables.

The correlation between epigenetic and chronological age was tested using Pearson’s product-moment correlation. The analysis was performed for the entire sample and stratified by tissue used for epigenetic age estimation (blood or saliva).

To assess the epigenetic age acceleration differences between the FHR and controls linear mixed-effects models were used, considering the FHR status as a dependent variable, epigenetic age acceleration as a fixed effect and family relatedness as a random effect. The analyses performed with the entire sample were corrected by sex and tissue used for epigenetic age estimation. The analyses stratified by tissue used for epigenetic age estimation were corrected by sex.

The effect of environmental factors (obstetric complications, socioeconomic statuses and recent stressful life events) on the epigenetic age acceleration was assessed using a linear mixed-effects model corrected for sex and the tissue used for the epigenetic age estimation, with family relatedness as a random effect.

Follow-up analysis using cell-adjusted epigenetic age acceleration estimates were performed. Therefore, similar linear mixed-effects models were constructed to evaluate differences between FHR and control groups and the effect of environmental factors.

## Results

### Sample description

One hundred seventeen children and adolescents aged 6–17 years (54.7% females) were included in this study, 53 of whom were at FHR (45.3%) and 64 were controls (54.7%). The main sociodemographic of the study sample and the differences between the FHR individuals and controls are shown in Table 1. Only the proportion of FHR individuals reporting an obstetric complication was found significantly increased (p.adj = 0.036). Age distribution of FHR and control individuals is shown in Supplementary Fig. 1.

**Table 1:** Summary and group comparison of the sociodemographic and diagnostic features of the sample (*n* = 117).

Feature	All (*n* = 117)	FHR (n = 53)	Control (*n* = 64)	Comparison	
	*n* (%) or mean (SD)			*t* or *χ*^2^	p.adj
Age	11.9 (3.2)	11.8 (3.1)	12.0 (3.2)	−0.275	0.784
Sex—female	64 (54.7%)	28 (52.8%)	36 (56.2%)	0.033	0.071
Obstetric complications	30 (25.6%)	20 (37.7%)	10 (15.6%)	6.320	**0.036**
Socioeconomic status	51.4 (12.6)	48.2 (14.1)	53.9 (10.8)	−2.211	0.058
Recent stressful life events—number (z-score)	−0.1 (0.9)	−0.2 (0.9)	−0.1 (0.9)	−1.005	0.476
Recent stressful life events—impact (z-score)	0.0 (0.9)	−0.1 (0.9)	0.1 (0.9)	−0.999	0.384

Significant differences are marked in bold.

*FHR* familial high risk.

### Epigenetic age correlation with chronological age

**Fig. 1:**
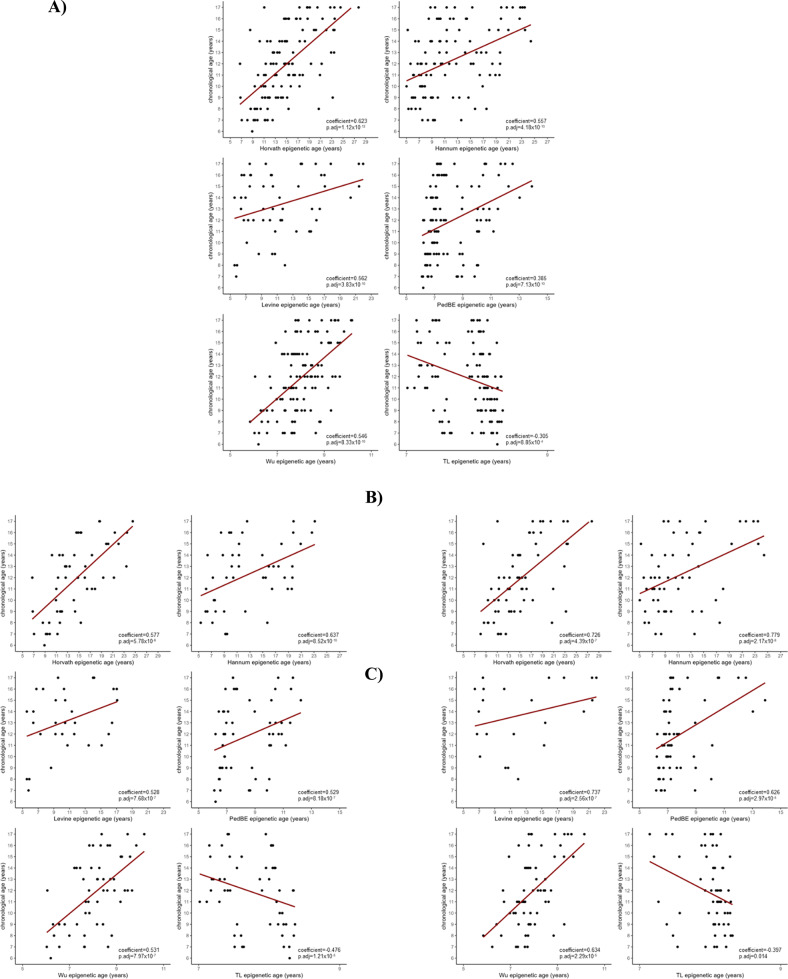
Linear correlation between chronological and epigenetic age. Epigenetic age estimated with the 6 epigenetic clocks was significantly correlated with correlated age in **A** the whole sample, **B** the individuals who provided blood and **C** the individuals who provided saliva for methylation analysis. Correlation was assessed with Pearson’s correation coefficient.

The chronological age of the sample was consistently correlated with the estimated epigenetic ages calculated with the epigenetic clocks, and inversely correlated with the estimated TL (p.adj < 0.005 for all analyses) (Fig. 1A). Significant correlations were obtained after the stratification of the sample by tissue used for epigenetic age estimation (p.adj < 0.005 for all analyses) (Fig. 1B, C; Supplementary Table 1).

### Epigenetic age acceleration and FHR

**Fig. 2:**
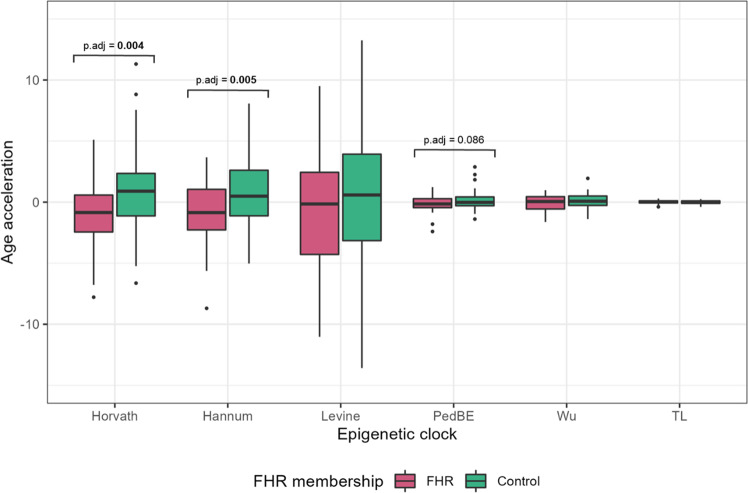
Boxplots showing epigenetic age acceleration differences between FHR and control individuals. TL telomere length, FHR familial high risk.

Epigenetic age acceleration differences between FHR and control groups were assessed for all the epigenetic clocks. The analyses showed that FHR individuals had a deceleration in the epigenetic ages estimated by three epigenetic clocks, although only two of them survived multiple testing correction. Specifically, the FHR individuals reported epigenetic age negative acceleration for the Horvath and Hannum epigenetic clocks (p.adj = 0.004, p.adj = 0.005) and a trend was found for PedBE (p.adj = 0.086) (Fig. 2). To check for tissue-specific differences between the FHR and control groups, the analyses were stratified by blood and saliva tissues. No epigenetic age acceleration was found different for any of the tissues (p.adj > 0.05 for all analyses) (Supplementary Table 2).

### Environmental risk effect on the epigenetic age acceleration

The effect of obstetric complications, the socioeconomic statuses and recent stressful life events on the epigenetic age acceleration was assessed. No significant effect of the environmental factors was found to be associated with the epigenetic age acceleration (p.adj > 0.05 for all the analyses) (Table 2).

**Table 2:** Effects of obstetric complications, socioeconomic status and recent stressful life events on the epigenetic age acceleration.

Epigenetic age acceleration	Obstetric complications			Socioeconomic status			Recent stressful life events (number)			Recent stressful life events (impact)		
	*t*	*R*^2^	p.adj	*t*	*R*^2^	p.adj	*t*	*R*^2^	p.adj	*t*	*R*^2^	p.adj
Horvath	0.286	0.001	1.000	−0.476	0.002	1.000	−0.438	0.004	0.994	−0.211	0.002	0.953
Hannum	1.012	0.009	0.837	0.029	0.004	0.977	−0.515	0.000	1.000	−0.235	0.010	1.000
Levine	1.582	0.021	1.000	−1.453	0.022	1.000	−1.263	0.017	1.000	−1.174	0.015	0.834
PedBE	0.197	0.000	0.921	1.000	0.000	0.767	−0.559	0.004	1.000	−0.396	0.002	0.978
Wu	1.777	0.026	1.000	−0.483	0.003	1.000	−1.024	0.009	0.925	−0.718	0.004	1.000
TL	0.046	0.000	1.000	−0.221	−0.001	0.991	1.193	0.013	0.943	1.224	0.014	1.000

*TL* telomere length.

### Cell-adjusted epigenetic age acceleration analyses

Differences between FHR and control groups and environmental risk effect on epigenetic age acceleration were assessed with the cell-adjusted epigenetic age acceleration measures. FHR individuals reported epigenetic age negative acceleration for the Horvath, Hannum and PedBE epigenetic clocks (p.adj = 0.013, p.adj = 0.020, p.adj = 0.035; respectively). No environmental risk factors had a significant effect on any epigenetic clock (p.adj > 0.05 for all analyses) (Supplementary Table 3).

## Discussion

The early stages of psychiatric disorders play a critical role in prognosis and outcome. Thus, the identification and characterization of risk factors and their molecular repercussion are key to understanding the mechanisms underlying psychopathology [51]. In this study, we characterized the epigenetic age of the young offspring of patients with schizophrenia and bipolar disorder. Compared to the offspring of control individuals, the FHR individuals reported a deacceleration of their epigenetic age relative to their chronological age for the Horvath and Hannum epigenetic clocks and no differences for the PedBE, Levine, Wu and TL clocks. None of the environmental stressors included could be associated with this phenomenon. Our results suggest that individuals at high risk may present epigenetic decelerated aging, which is in accordance to previous findings but may conflict with the accelerated aging hypothesis in schizophrenia.

Methylation data were extracted from blood and saliva samples. Although Horvath epigenetic clock can estimate the epigenetic age accurately in most tissues, the rest of epigenetic clocks perform best in either blood or saliva samples. The consistent correlation of epigenetic and chronological ages in the whole sample and in both tissues separately implies that in this particular case, variation generated by the DNA source used for methylation does not have a great impact. Yet, all analyses were performed using methylation tissue as a covariate to minimize any possible effect.

Positive acceleration of the epigenetic age reflects premature cellular aging, while negative acceleration—i.e. deacceleration—denotes a slower pace of cellular aging. We identified epigenetic deacceleration in the FHR individuals for the Horvath, Hannum and PedBE epigenetic clocks, although the latter did not survive multiple testing correction. No tissue-specific differences in epigenetic age acceleration were found, possibly due to the sample size reduction and subsequent loss of statistical power. We found trends towards signification for Horvath and Hannum epigenetic clocks in blood and saliva analyses, suggesting that the tissue used for epigenetic age estimation was not a critical factor.

Previous studies in schizophrenia and bipolar disorder adult samples using epigenetic clocks based on age-related methylation markers—i.e. Horvath, Hannum, PedBE and Wu—have found inconsistent results. Four studies reported epigenetic deacceleration for at least one of the clocks included in the analyses [52–54]—one only in schizophrenia males treated with clozapine [9]—, four did not detect significant differences for epigenetic age acceleration [9, 55–60] and two detected epigenetic age acceleration only in older bipolar disorder patients [61] and a small acceleration in schizophrenia patients [9]. As for the Levine clock/phenotypic age, accelerated epigenetic aging [9, 59] and no differences with chronological age [60] have been found, providing no conclusive distinction between chronologic and phenotypic age epigenetic clocks. The epigenetic clock for TL found shorter lengths—thus implying aging acceleration—in schizophrenia patients [9]. Two studies analyzed longitudinally the effect of first-episode psychosis on epigenetic age acceleration measured with Horvath clock, reporting deaccelerated epigenetic ages before psychosis onset and increased acceleration rates after the psychotic episode and the exposition to antipsychotic medication [62, 63]. Therefore, these previous findings suggest that each epigenetic clock could reflect diverse aspects of aging and that the pace of epigenetic aging could be irregular along the lifespan, reporting more pronounced deviations from chronological age in certain stages of life [9, 61] and due to psychosis onset or exposition to pharmacological treatment. Thus, disorder stages—including the ones preceding the onset—have to be considered to establish the epigenetic age dysregulation as a molecular predictor on mental illness.

The exposition to environmental stressors has been thoroughly considered as a mediator of biological aging. Lifestyle, diet, substance use, education, economic income, psychosocial stress, disease and many other factors have been found to alter biological aging [64–67]. Biological aging has also been found to be affected by the prenatal environment and early life adversity [68–77]. Our results could not confirm the association of environmental stress exposure with a dysregulation of time-dependent methylation pattern. Several reasons may explain the lack of association between environmental stressors and the epigenetic age in our sample. The present sample was recruited at a very young age, making it difficult to detect the cumulative impact of environmental stressors. We lacked a measure covering all environmental stressors, and the use of discreet indexes may have limited our capacity to detect small changes. Moreover, our sample had a low frequency of obstetric complications and showed high homogeneity in the sociodemographic status and frequency of recent stressful life events. Further studies with larger samples and more refined tools for prospectively measuring the effect of environmental insults are required to define their role as mediators of epigenetic changes and to understand their contribution to psychopathology.

The analyses performed with cell-adjusted epigenetic age acceleration estimates in the entire sample reported identical results in FHR/control group differences (in exception of the PedBE epigenetic clock surviving multiple testing correction) and on the effect of environmental risk factors. The similarity of results suggests that the FHR condition or any of the studied environmental risk factors had a critical effect on blood/saliva cell proportions.

Some limitations of the present work should be taken into consideration. Firstly, the sample size was limited. Therefore, the statistical analysis may have been underpowered to detect small effects. Epigenetic characterization was conducted using heterogeneous biological samples, but parallel data processing was performed for the blood and saliva samples and all the analyses were corrected for tissue type. The study lacked the assessment of alcohol and tobacco use during pregnancy, a putatively confounding variable in epigenetic studies. Participants were recruited based on their family history of either schizophrenia or bipolar disorder, which could have contributed additional heterogeneity in terms of determinants of risk. Finally, the young age of the participants impeded the categorization of the subjects based on their conversion to bipolar disorder or schizophrenia, a presumably more homogeneous phenotype. Nonetheless, the current definition based on familial risk has so far proven to be valid for detecting differences in psychopathology as well as in neuropsychological and brain imaging features [30, 78–84].

Molecular mechanisms driven by epigenetic changes in early stages of life may be critical for the onset of severe mental disorders and their clinical course. The epigenetic age asynchronicities found in the young at high risk provide novel evidence to advance towards the characterization of the molecular signature driven by environmental stressors. The effects of a discordant pace in biological aging could be critical to understand the underlying mechanisms of illness onset and the age-associated conditions detected in schizophrenia and bipolar disorder. Further studies are required to identify the relevance, causality and interaction of internal and external elements that define the clinical manifestation of severe mental disorders to ultimately develop novel tools for personalized psychiatry.

## Supplementary information

Supplemental Material

## Data Availability

According to the permiss ions of the informed consent, clinical and biological data of the participants of the studies cannot be accessed through public repositories. Epigenetic data results, such as epigenetic age estimation, can be shared upon request. The authors confirm that the data supporting the findings of this study are available within the article and its supplementary materials. *methylclock* R package documentation can be found at https://github.com/isglobal-brge/methylclock. For cell count estimation, publicly available data were used for blood [48] and saliva [49] tissues.

## Code availability

The code for methylation data preparation, epigenetic clock construction and statistical analysis is not publicly available and can be obtained upon request.
